# Investigating the Potential Involvement of Glutathione S-Transferases in Defence Against Powdery Scab in Potato

**DOI:** 10.3390/jof11090654

**Published:** 2025-09-04

**Authors:** Sadegh Balotf, Richard Wilson, Calum Wilson

**Affiliations:** 1 Centre for Crop Health, University of Southern Queensland, Toowoomba, QLD 4350, Australia; 2 Central Science Laboratory, University of Tasmania, Hobart, TAS 7001, Australia; richard.wilson@utas.edu.au; 3 Tasmanian Institute of Agriculture, University of Tasmania, Hobart, TAS 7005, Australia; calum.wilson@utas.edu.au

**Keywords:** glutathione S-transferases, potato, *Spongospora subterranea*, transcriptomics, proteomics

## Abstract

Glutathione S-transferases (GSTs) are key enzymes in plant defences involved in detoxification, redox regulation, and the modulation of secondary metabolism, playing essential roles in the response to pathogen infections. Despite prior genome-wide predictions of GSTs in potato, little is known about their specific roles in defending against *Spongospora subterranea*. This study re-analyses our previously generated transcriptomics and proteomics datasets to explore the role of GSTs in two contrasting potato cultivars, ‘Iwa’ (susceptible) and ‘Gladiator’ (resistant), after inoculation with *S. subterranea*. A total of 69 and 41 GSTs were identified in the transcriptomics and proteomics data, respectively. The majority of these GSTs were upregulated in the resistant cultivar but not in the susceptible cultivar. The upregulation of GSTs in ‘Gladiator’ suggests a more efficient antioxidant and detoxification response following *S. subterranea* infection. Chromosomal mapping revealed a high number of GSTs on chromosome 9, suggesting a hotspot for GSTs in the potato genome. This research provides direct evidence of the potential involvement of GSTs in resistance to *S. subterranea*, offering insights into potential targets for breeding resistant potato cultivars.

## 1. Introduction

*Spongospora subterranea*, the causative agent of powdery scab in potato (*Solanum tuberosum*), represents one of the most significant threats to potato production worldwide [1]. This soil-borne pathogen infects potato plants, leading to root dysfunction and tuber lesions, which reduce yield and degrade tuber quality [2]. In addition, *S. subterranea* also serves as a vector for potato mop-top virus, another major pathogen that compromises potato productivity further [3]. The lifecycle of *S. subterranea* is complex and highly specialised, requiring a living host to complete its development. As a biotrophic pathogen, the interaction between the pathogen and the host is intimate [4]. Given the limited effectiveness of existing management strategies and the economic impact of this disease, the development of potato cultivars resistant to *S. subterranea* is critical [5]. Such resistance could provide a sustainable means of managing the disease, but achieving it requires a deeper understanding of defence mechanisms during infection by *S. subterranea* in potato.

Plant-specific glutathione S-transferases (GSTs) are a class of multifunctional enzymes that play essential roles in the defence against pathogen infections [6]. GSTs are involved in a wide range of cellular processes, such as detoxification, redox regulation, and the modulation of secondary metabolism. Large and diverse gene families encode these enzymes and are often upregulated in response to environmental stresses [7]. GSTs act as antioxidants and reduce the severity of infection symptoms by limiting pathogen levels within plant tissues [7]. They achieve this by catalysing the conjugation of glutathione to electrophilic or hydrophobic substrates, thereby detoxifying the harmful compounds produced during pathogen invasion [8]. GSTs also play an important role in the plant stress signalling network, interacting with key molecules such as reactive oxygen species (ROS) and salicylic acid, both of which are central to the plant’s defence response [9]. While many studies show that GSTs help plants to resist pathogens, other research suggests that some GSTs may not play a protective role. In tea plants, CsGSTU45, a specific GST gene, promotes susceptibility to *Colletotrichum camelliae* infection through the jasmonate signalling pathway [10]. These contrasting examples show that the role of different GSTs in plant resistance to pathogens is complex and not yet fully understood.

Recent genome-wide studies have predicted several GSTs in the potato genome. One such study identified 90 GSTs in the potato genome based on homology/predicted function and undertook an expression analysis of these GSTs based on expression values retrieved from other studies [11], while another study predicted 366 GSTs, of which 16 responded to both drought stress and DNA demethylation based on a transcriptomics analysis in potato [12]. These studies have mostly been based on genome-wide analyses, with some having partial support from transcriptomics, and yet, little is known about the expression of these GSTs in response to pathogen infection at different levels, including the RNA and protein levels.

Transcriptomics enables the identification of differentially expressed genes by providing a global view of the gene expression changes during infection [13]. Proteomics, on the other hand, complements transcriptomic data by identifying and quantifying the actual proteins expressed in plant tissues, including post-translational modifications, which can affect protein function and stability [14]. Importantly, a proteomic analysis helps to confirm whether transcriptional changes are translated into functional outcomes at the protein level. When combined, these omics approaches offer a systems-level understanding of plant–pathogen interactions and enable the identification of key molecular players involved in resistance responses [15].

Given the challenges posed by powdery scab, it is essential to establish a comprehensive dataset that encompasses the full spectrum of potential GSTs and to define those involved in conferring resistance to *S. subterranea* infection better. This study aims to explore the role of GSTs in the potato–*S. subterranea* interaction by analysing the transcriptomic and proteomic datasets generated in our previous study [16]. Specifically, we seek to identify GST family members that may contribute to resistance mechanisms, thereby providing a foundation for future functional studies and breeding strategies.

## 2. Materials and Methods

The current study used the transcriptomic and proteomic datasets generated in Balotf et al. [16]. The following protocol provides a summary of the experimental procedures to support the data interpretation.

### 2.1. Plant Materials

The potato cultivars ‘Gladiator’ and ‘Iwa’ used in this study were generously provided by New Zealand Plant & Food Research(Auckland, New Zealand), the owners of these varieties. The cultivar ‘Gladiator’ exhibits strong resistance to both tuber and root diseases caused by *S. subterranea*, while the cultivar ‘Iwa’ is highly susceptible to diseases caused by this pathogen [17,18]. Potato plants were propagated from single-node cuttings and grown under sterile tissue culture conditions on Murashige and Skoog medium supplemented with 30 g/L of sucrose, 500 mg/L of casein hydrolysate, and 40 mg/L of ascorbic acid. During the initial growth stage, the plants were maintained at 22 °C under a 16 h light/8 h dark photoperiod.

### 2.2. The Preparation of S. subterranea Inoculum and the Root Infection Procedure

Resting spores of *S. subterranea* were obtained from powdery-scab-infected potato tubers collected from a commercial potato field in Tasmania, Australia. The presence of *S. subterranea* in the collected lesions was confirmed through PCR using species-specific primers, as previously described [19]. To stimulate germination, 2 mg of dried spore material, containing approximately 20,000 sporosori per milligram (estimated using hemocytometer counts from diluted spore suspensions), was suspended in 2 mL of Hoagland solution (HS) and incubated at 25 °C for 72 h. Tissue-cultured plantlets (~8 cm tall) from each potato cultivar were selected for inoculation, with their roots immersed in the spore suspension for one hour. An equal number of control plants was inoculated using sterile water. All plants were then transplanted into 2 L pots containing sterilised potting mix composed of sand, loam, and composted pine bark. To maintain a consistent infection pressure, 20 mL of freshly prepared spore suspension was applied to each pot two weeks after transplanting. The plants were grown under controlled greenhouse conditions (25 ± 3 °C, 80 ± 5% relative humidity, 16 h photoperiod). After 42 days, roots from both the inoculated and control plants were harvested; thoroughly washed; snap-frozen in liquid nitrogen; and stored at −80 °C. The transcriptomics analysis was performed using three biological replicates (one plant per replicate), while the proteomics analysis was conducted using four biological replicates.

### 2.3. The Transcriptomics and Proteomics Experiments

Total RNA was extracted from 50 mg of frozen roots using the RNeasy Plus Mini Kit (Qiagen, Hilden, Germany), and genomic DNA was removed using the gDNA Eliminator spin columns (Qiagen, Hilden, Germany). RNA quantity and quality were assessed using a Qubit fluorometer (Invitrogen, Waltham, MA, USA) and an Agilent 2100 Bioanalyzer system (Agilent, Palo Alto, Santa Clara, CA, USA). mRNA-seq libraries were constructed using the TruSeq Stranded Total RNA Library Prep Kit with RiboZero (Illumina, San Diego, CA, USA), and sequencing was performed on the NovaSeq6000 platform at the Australian Genome Research Facility (AGRF, Melbourne, VIC, Australia). For proteomics, 50 mg of frozen root tissue per sample was homogenised, and proteins were extracted using a denaturing buffer including 7 M urea and 2 M thiourea, 100 mM NaCl, 1% dithiothreitol (DTT), 40 mM Tris, a pH of 8.0, and a protease inhibitor cocktail (cOmplete Mini EDTA-free; Roche Diagnostics, Sydney, NSW, Australia). The protein samples were reduced and alkylated before being digested using the SP3 protocol [20] with trypsin/LysC (Promega, Madison, WI, USA). Peptides were desalted and analysed using nanoLC-MS/MS on a Q-Exactive HF mass spectrometer (Thermo Fisher Scientific, Waltham, MA, USA) coupled with an Ultimate 3000 nanoHPLC system (Thermo Fisher Scientific, Waltham, MA, USA), following the procedure previously described by Balotf et al. [21].

### 2.4. Data Analysis

For the RNA sequencing data, quality control of the raw reads was conducted with FastQC, and adapter contamination was removed using Trimmomatic v.0.36.6 [22]. High-quality RNA-seq reads were aligned with the potato genome [23] using the HISAT2 v.2.1.0 tool [24]. A count matrix of the uniquely mapped fragments per gene was generated using FeatureCountsv.2.0.1, and a differential gene expression analysis was performed using DESeq2v.2.11.40.6, with a false discovery rate (FDR)-adjusted *p*-value cut-off of 0.05. The proteomics data were processed with Spectronaut software (v 14.7), and the protein analysis was performed using Perseus software version 1.6.14.0 [25]. The functional analysis of identified GSTs was conducted in ShinyGO v0.82, with “*Solanum tuberosum* L. (DM_1-3_516_R44 v6.1)—Doubled Monoploid Potato” as the reference genome and all of the other settings kept at the default [26].

## 3. Results and Discussion

### 3.1. Identification of GSTs in the Transcriptomics and Proteomics Data

**Figure 1:**
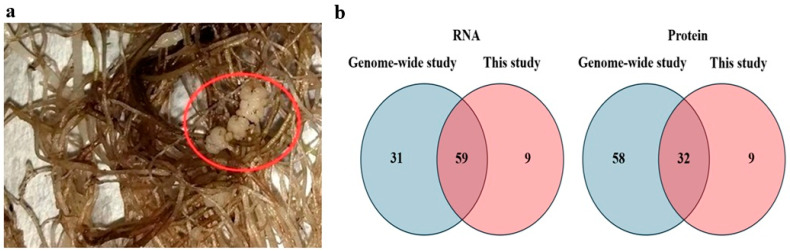
*Spongospora subterranea*-infected potato roots with root galls in the powdery-scab-susceptible cultivar ‘Iwa’. Galls formed approximately six weeks after the initial inoculation of the potato roots with *S. subterranea* (**a**). The number of identified GSTs in our study compared to a previous comprehensive genome-wide analysis by Islam et al. [11]. In both the transcriptomics and proteomics analyses of the potato roots infected with *S. subterranea*, new GSTs that had not been reported in genome-wide analyses were identified (**b**).

Susceptible and resistant potato cultivars were inoculated with *S. subterranea*, and after 42 days, when root galls were visible (Figure 1a), roots were collected for the transcriptome and proteome analyses. It is worth noting that according to previous studies, no potato cultivar is completely resistant to *S. subterranea*, and even resistant varieties such as ‘Gladiator’ can become infected and develop root galls under conducive conditions [17]. However, the number and size of the galls were significantly greater in the susceptible cultivar compared to those in the resistant cultivar. The number of GSTs identified in our study was then compared to a previous comprehensive genome-wide analysis undertaken by Islam et al. [11]. In both the transcriptomics and proteomics analyses of the potato roots infected with *S. subterranea*, new GSTs that had not been reported in genome-wide analyses were identified (Figure 1b). Additional information regarding the GSTs identified in this study is presented in Supplementary File S1.

**Figure 2:**
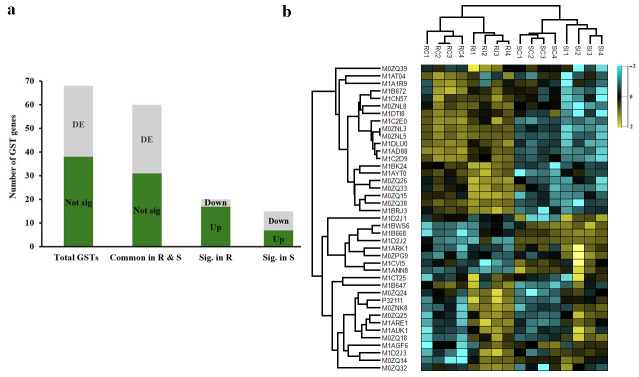
Total identified and differentially expressed (DE) GSTs (FDR < 0.05) in the transcriptome analysis of resistant (R) and susceptible (S) potato cultivars infected with *S. subterranea* (**a**). Almost half of the identified GST genes were significantly altered after *S. subterranea* infection, with more GSTs being upregulated in the resistant cultivar and more being downregulated in the susceptible cultivar. The Z-scored abundance of identified GSTs in the proteomics analysis of resistant (R) and susceptible (S) potato cultivars infected with *S. subterranea* (**b**). RC: resistant control; RI: resistant infected; SC: susceptible control; SI: susceptible infected.

In the transcriptomics data, 68 GSTs were identified, with nearly half being differentially expressed following infection by *S. subterranea* (Figure 2a). Interestingly, out of 30 differentially expressed GSTs, 17 were upregulated in the resistant cultivar ‘Gladiator’, while in the susceptible cultivar ‘Iwa’, most of these genes were downregulated (Figure 2a). A similar pattern was observed in the proteome analysis, where 41 GSTs were identified, 13 of which were upregulated in the resistant cultivar, and only 1 was downregulated (FDR < 0.05). In contrast, in the susceptible cultivar, the levels of these enzymes were not significantly altered, with only five being upregulated (Figure 2b). Across both datasets, 30 GSTs were shared, resulting in a total of 79 unique GSTs identified in this study (Supplementary File S1). The differential expression of the GSTs observed between ‘Gladiator’ and ‘Iwa’ underscores the likely involvement of GSTs in the defence response to *S. subterranea*. GSTs play crucial roles in plant defence, particularly through the detoxification of ROS and the conjugation of toxic compounds with glutathione, aiding in their sequestration and removal [6,27]. During pathogen infection, ROS are rapidly produced as part of the oxidative burst, a key component of basal and induced immunity [28,29]. While ROS act as signalling molecules to activate downstream defence responses, their uncontrolled accumulation can lead to oxidative damage [30]. Therefore, timely induction of detoxifying enzymes like GSTs is essential for maintaining redox homeostasis and allowing effective immune signalling without incurring cellular injury.

In this study, the upregulation of nearly half of the identified GSTs in ‘Gladiator’ suggests a more efficient antioxidant and detoxification response following *S. subterranea* infection. Moreover, GSTs have also been implicated in the regulation of phytohormone signalling pathways, such as those involving salicylic acid and jasmonic acid, hormones known to be integral to plant immunity [31,32]. Their upregulation in ‘Gladiator’ may therefore also reflect broader transcriptional reprogramming toward a defensive physiological state. In contrast, the downregulation of most of the GSTs in ‘Iwa’ indicates a weaker stress response and may suggest an inability to adequately control oxidative stress or mobilise hormonal defences, potentially contributing to its susceptibility [33].

### 3.2. The Functional Analysis of the Identified GSTs

**Figure 3:**
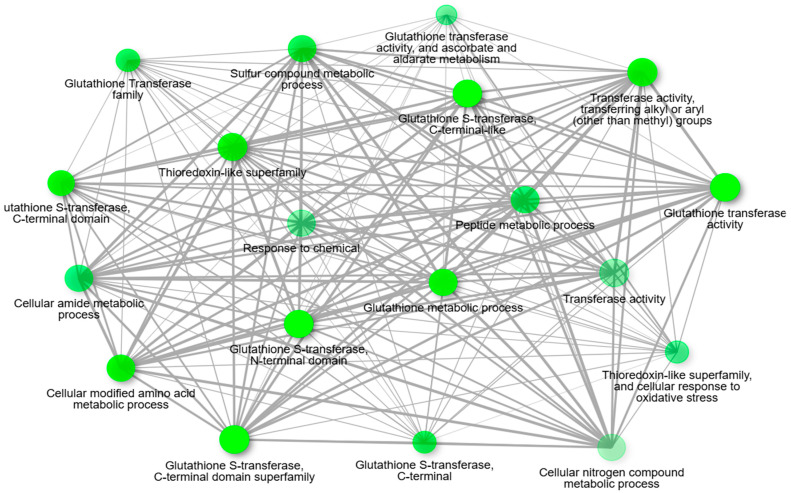
The association network analysis of the enriched pathway of identified GSTs. Darker nodes (more intense in colour) are more significantly enriched gene sets, and bigger nodes represent larger gene sets. Thicker edges represent more overlapped genes.

To investigate the molecular response to *S. subterranea* infection further, we performed an association network analysis (Figure 3) that integrated both the transcriptomic and proteomic data to map all identified GSTs. This analysis provided insights into the broader regulatory and functional interactions involving GSTs during infection. In addition, we classified GSTs that were specifically upregulated in the resistant cultivar, as summarised in Table 1. The functional analysis revealed that upregulated GSTs are involved in several biological processes, with the most significant ones being “Glutathione metabolic process” and “Amino acid metabolic process”. The involvement of GSTs in processes like glutathione conjugation further emphasises their importance in cellular protection, maintenance of redox balance, and the modulation of various signalling pathways essential for cell survival and adaptation. The functional enrichment analysis of the GSTs upregulated in ‘Gladiator’ reinforces their central role in the plant’s defence strategy against *S. subterranea*. The significant association of these GSTs with the “glutathione metabolic process” is consistent with their known function in detoxifying electrophilic compounds through conjugation with glutathione [27,34]. This pathway not only aids in the neutralisation and compartmentalisation of toxic metabolites generated during pathogen attack but also contributes to maintaining cellular redox homeostasis, which is essential for activating and regulating defence signalling cascades [35]. The observed enrichment of GSTs in the “amino acid metabolic process” further suggests that these enzymes may be involved in adjusting primary metabolism to support defence responses [36]. Amino acids serve as precursors for numerous secondary metabolites, including phytoalexins and signalling molecules, which are instrumental in pathogen resistance [37].

### 3.3. Chromosomal Localisation of GSTs in the Potato Genome

**Figure 4:**
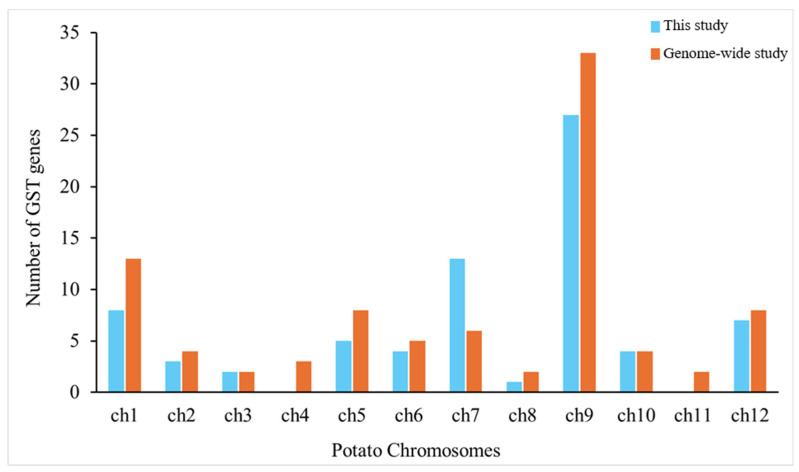
The distribution of 79 identified GSTs on 12 different chromosomes of potato. The bar graph with light blue bars shows the distribution of identified GSTs across potato chromosomes in our study, while the orange bars show the identified GSTs in a previous genome-wide study. Both studies indicate that chromosome 9 may represent a potential hotspot for GST genes in the potato genome.

To explore the genomic distribution of the GST genes identified in our study, we mapped them across the 12 chromosomes of the potato genome [23]. For comparison, we also mapped the 90 GSTs previously reported by Islam et al. [11]. Both studies revealed a notable concentration of GSTs on chromosome 9, making it a promising target for further investigation through quantitative trait locus (QTL) mapping, as it may contain key genetic loci involved in the regulation of GST activity and associated resistance traits [38,39]. Despite this common finding, differences in chromosomal distribution were observed. While the previous study identified GSTs on all 12 chromosomes of the potato, our study did not detect any GSTs on chromosomes 4 and 11 (Figure 4). This discrepancy suggests that potato plants may utilise different GSTs located on different chromosomes in response to various stresses, highlighting the possibility of stress-specific gene regulation and chromosomal distribution [40,41]. The absence of GSTs on chromosomes 4 and 11 in our study could also reflect cultivar-specific variations or limitations within the transcriptomics and proteomics datasets used, which were based on a single time point. It is possible that potato plants could upregulate other GSTs at different time points, such as during early infection.

## 4. Conclusions

In conclusion, although previous studies have identified several GSTs in potato based on genome-wide predictions, our research comparing resistant vs. susceptible potato cultivars is the first to provide direct evidence of their potential role in resistance to *S. subterranea*. By integrating data at both the RNA and protein levels, we were able to identify specific GST isoforms that are differentially expressed in resistant cultivars, thereby reinforcing their functional relevance in pathogen defence. Building on previous insights from other species, such as the role of GST theta 2 in activating systemic acquired resistance via epigenetic mechanisms in *Arabidopsis thaliana* [42], our study underscores the functions of GSTs in plant immunity. Additionally, transcriptomic analyses in *Populus tomentosa* [43] have similarly demonstrated an elevated GST expression in response to pathogen infestation, suggesting conserved roles across species. Furthermore, functional validation studies like those involving the tau class GST LrGSTU5 in *Lilium regale*, which enhanced the resistance against *Fusarium oxysporum* in transgenic tobacco [44], highlight the potential of specific GSTs to improve disease resistance when manipulated.

Our data revealed a wide range of GST isoforms expressed in potato roots during *S. subterranea* infection, with marked upregulation in the resistant cultivar, which likely supports the more effective detoxification of reactive oxygen species and pathogen-derived toxins [45]. Functional enrichment analyses linked these GSTs to glutathione metabolism and amino acid pathways, emphasising their role in maintaining redox balance and supporting defence-related metabolic adjustments [46]. The clustering of GST genes on chromosome 9 points to a genomic hotspot that may contain key resistance loci, providing promising targets for breeding efforts.

Our findings provide a foundation for future research focused on understanding the exact roles of different GST isoenzymes in enhancing potato’s resistance to powdery scab disease. Functional validation studies, such as gene knockouts or overexpression assays, will be crucial for confirming the specific contributions of individual GSTs. Future experiments, including the analysis of additional cultivars and infection time points, are needed to elucidate the precise role of GSTs in the defence against *S. subterranea* in potato further. Once these roles are fully understood, this knowledge could be incorporated into breeding programmes aimed at developing potato cultivars with enhanced resistance to disease caused by *S. subterranea*. Furthermore, the use of beneficial bacteria to treat plant roots has been shown to enhance resistance through the modulation of plant defence pathways by increasing specific GSTs [47]. Such approaches could offer a complementary strategy to boost the resistance against powdery scab and may represent an effective component of integrated disease management strategies.

## Figures and Tables

**Table 1:** The functional analysis of GSTs upregulated in the resistant cultivar.

FDR	nGenes	Fold Enrichment	Pathways
1.1 × 10^−55^	21	501.8	Glutathione metabolic process
9.2 × 10^−51^	21	301.9	Amino acid metabolic process
1.0 × 10^−40^	21	104.5	Sulfur compound metabolic process
8.0 × 10^−34^	21	49.1	Peptide metabolic process
4.5 × 10^−32^	21	40.2	Cellular amide metabolic process
2.5 × 10^−25^	22	15.7	Response to chemical
8.1 × 10^−19^	21	9.3	Cellular nitrogen compound metabolic process

## Data Availability

The data used in this paper were made available through our previously published work [16] with the following accession numbers: The RNA-seq raw reads are available via the NCBI-SRA database under BioProject PRJNA776331. The MS/MS raw data were deposited to the ProteomeXchange Consortium via the PRIDE partner repository with the dataset identifier PXD029381.

## Supplementary Materials

The following supporting information can be downloaded at https://www.mdpi.com/article/10.3390/jof11090654/s1. File S1: List of identified GSTs in our study.

## Abbreviations

The following abbreviations are used in this manuscript:

DE	Differentially expressed
DTT	Dithiothreitol
EDTA	Ethylenediaminetetraacetic acid
FDR	False discovery rate
GSTs	Glutathione S-transferases
HPLC	High-performance liquid chromatography
HS	Hoagland solution
MS	Mass spectrometry
QTL	Quantitative trait locus
ROS	Reactive oxygen species
